# INTEGRATING A PATIENT-CENTERED, WHOLE-HEALTH APPROACH WHEN CARING FOR PEOPLE AGING WITH HIV

**DOI:** 10.1093/geroni/igad104.0379

**Published:** 2023-12-21

**Authors:** Gemmae Fix, Janet Clark, Allen Gifford

**Affiliations:** US Department of Veteran Affairs, Bedford, Massachusetts, United States; VA Office of Patient Centered Care & Cultural Transformation, Washington, District of Columbia, United States; VA Center for Healthcare Organization & Implementation Research, Boston, Massachusetts, United States

## Abstract

The US Department of Veteran Affairs (VA) is implementing a patient-centered, “Whole Health System of Care” to align care with What Matters Most to patients within their unique circumstances. Implementation is focused on primary care, where most patients receive care. Yet HIV Clinics have historically provided both HIV and primary care because of the complexity of HIV. People living with HIV are now aging and experiencing a constellation of health and life circumstances that would benefit from a Whole Health approach. While there is desire to spread Whole Health beyond primary care, little is known about how to integrate Whole Health into HIV Clinics. We sought to identify Whole Health implementation considerations for HIV Clinics. Our qualitative study design included interviews with vignettes based on our prior work, illustrating themes like aging alone. Findings were mapped to the i-PARIHS implementation framework which includes recipients (HIV providers), context (HIV clinics), and innovation (Whole Health). We interviewed physicians, psychologists, nurses and leadership (N=11) from three HIV Clinics (Northeast, Northwest, South). Participants were largely aware of Whole Health, although few had taken trainings and no clinics currently offered Whole Health services. Many expressed interest incorporating Whole Health into HIV care. They also saw great value, given patients’ complexities. Participants described logistical barriers hindering implementation, like awareness of specific resources or how to refer. Only some providers were able to conceptualize a Whole Health clinical encounter- where patients’ life circumstances are connected to health. HIV Clinics offer an ideal setting to spread Whole Health.

